# Investigation of the Influence of Wound-Treatment-Relevant Buffer Systems on the Colloidal and Optical Properties of Gold Nanoparticles

**DOI:** 10.3390/nano13121878

**Published:** 2023-06-17

**Authors:** Atiđa Selmani, Ramona Jeitler, Michael Auinger, Carolin Tetyczka, Peter Banzer, Brian Kantor, Gerd Leitinger, Eva Roblegg

**Affiliations:** 1 Pharmaceutical Technology & Biopharmacy, Institute of Pharmaceutical Sciences, University of Graz, Universitätsplatz 1, 8010 Graz, Austria; atida.selmani@uni-graz.at (A.S.); ramona.jeitler@uni-graz.at (R.J.); 2 Research Center Pharmaceutical Engineering GmbH, Inffeldgasse 13, 8010 Graz, Austria; carolin.tetyczka@rcpe.at; 3 Institute of Chemical Technologies and Analytics, TU Wien, Getreidemarkt 9/164, 1060 Vienna, Austria; michael.auinger@tuwien.ac.at; 4 Institute of Physics, NAWI Graz, University of Graz, Universitätsplatz 5, 8010 Graz, Austria; peter.banzer@uni-graz.at (P.B.); brian.kantor@uni-graz.at (B.K.); 5 Division of Cell Biology, Histology and Embryology, Gottfried Schatz Research Center for Cell Signaling, Metabolism and Aging, Medical University of Graz, 8010 Graz, Austria; gerd.leitinger@medunigraz.at

**Keywords:** gold nanoparticles, pH, buffer, ionic strength, size, zeta potential, wound healing

## Abstract

Biocompatible gold nanoparticles (AuNPs) are used in wound healing due to their radical scavenging activity. They shorten wound healing time by, for example, improving re-epithelialization and promoting the formation of new connective tissue. Another approach that promotes wound healing through cell proliferation while inhibiting bacterial growth is an acidic microenvironment, which can be achieved with acid-forming buffers. Accordingly, a combination of these two approaches appears promising and is the focus of the present study. Here, 18 nm and 56 nm gold NP (Au) were prepared with Turkevich reduction synthesis using design-of-experiments methodology, and the influence of pH and ionic strength on their behaviour was investigated. The citrate buffer had a pronounced effect on the stability of AuNPs due to the more complex intermolecular interactions, which was also confirmed by the changes in optical properties. In contrast, AuNPs dispersed in lactate and phosphate buffer were stable at therapeutically relevant ionic strength, regardless of their size. Simulation of the local pH distribution near the particle surface also showed a steep pH gradient for particles smaller than 100 nm. This suggests that the healing potential is further enhanced by a more acidic environment at the particle surface, making this strategy a promising approach.

## 1. Introduction

The microenvironment in the human body is highly complex in a healthy state and can change drastically in a diseased state. This is particularly the case with the skin, considering the progression from injury to wound healing. Wound healing can be classified into four integrated and partly overlapping phases, namely haemostasis, inflammation, proliferation and connective tissue remodelling to regenerate the functional epidermal barrier [1,2,3].

Thereby, many local wound factors and mediators are involved. One very important factor is the pH. If the skin, which shows a pH of 4–6 under homeostasis, is injured, there is initially increased lactic acid production and hypoxia before the pH at the wound site rises. Acute wounds have a neutral pH ranging from 6.5 to 8.5. This should theoretically reduce buffer capacity, which is also the case in inflammatory skin diseases, although limited data are available [4]. When wound healing is delayed, conditions become alkaline, bacterial growth is promoted, and healing is prevented [5]. At this stage, the wound is known as chronic and has an alkaline pH of 7.2–8.9, which favours bacterial growth. During healing, physiological mechanisms begin to restore the acidic environment by adjusting the pH. This, in turn, triggers cellular processes to restore the epithelial barrier, influences enzyme activity, and adjusts oxygen tension in the wound, which in turn promotes fibroblast growth and collagen synthesis. Cell growth is more active in acidic microenvironments, which are associated with faster migration and proliferation behaviour, eventually leading to wound regeneration. Because of these highly complex conditions, healing wounds remains a major challenge in modern medicine. It is assumed that the enhanced cell proliferation under acidic conditions is probably closely related to the polarity and epithelial potential between injured and uninjured tissue. This is consistent with several studies reporting that acidifying wounds, e.g., with topical creams, gels, dressings or solutions including lactate, acetic acid or citrate, is an effective treatment and also helps to bypass the alkaline microenvironment that promotes bacterial growth [6]. Besides buffered therapeutic systems, nanoparticles (NPs) have become increasingly interesting for therapeutic and diagnostic purposes in wound healing. The NPs used include inorganic/metallic NPs, and lipid- and polymer-based NPs [7]. While the latter are mainly used for drug delivery of enzymatically prone drugs that should be released in a controlled manner, metallic/inorganic NPs may exhibit intrinsic properties in addition to drug delivery, such as antimicrobial activity, anti-inflammatory activity, proangiogenic and antioxidant activity and optical activity. Gold (Au) NPs, for example, are versatile nanomaterials with unique physicochemical and optical properties that are biocompatible and can be fabricated in variable sizes and shapes [8,9]. Due to their excellent physicochemical and optical properties and nontoxic nature, AuNPs exhibit tremendous potential in the biomedical field, such as bio-imaging, diagnostics, photo-induced cancer therapy, tissue engineering and immunology [10]. The optical properties offer enormous opportunities for biosensor applications [11,12], as they exhibit a strong surface plasmon resonance (SPR) absorption band and a high molar absorptivity [13]. Their radical scavenging activity makes them attractive for wound therapy; likewise, they have been found to shorten wound healing time by, for example, improving re-epithelialization and promoting new connective tissue and microscopic blood vessel formation and extracellular matrix deposition. Unlike silver NPs, AuNPs do not possess antimicrobial activity themselves, but they allow efficient coupling with antimicrobial biomolecules and drugs, enhancing the effectiveness against microbes [14,15,16]. Moreover, they are modifiable and thus suitable for the targeted drug delivery of biomolecules such as DNA, RNA and proteins to, e.g., specific cells. This can be achieved with adsorption or through ionic or covalent binding with the help of linkers. Thereby, drug release can be controlled by various internal (e.g., pH value and enzymes) and external (e.g., light) stimuli, which, again, highlights their enormous potential [17,18,19].

To take advantage of wound acidification and the intrinsic properties of AuNPs, both strategies should be combined, i.e., AuNPs should be incorporated into different buffer systems relevant to wound treatment. In this respect, the influence of pH and ionic strength (*I_c_*) must be carefully considered, as it is known that pH-induced changes in surface chemistry or fluctuations in electrolyte levels lead to reduced NP stability and, consequently, the altered binding of cellular components or biomolecules [20]. This, in turn, affects cellular interactions, transport mechanisms, accumulation and/or excretion of the therapeutic potential of NPs [21,22]. In addition, local pH shifts caused by chemical reactions on the particle surface must be considered, as these might have a strong impact on the local environment [23] due to stronger pH gradients around smaller NPs [24]. However, steep pH gradients between the dispersant and the particle surface would further acidify the NP environment, which could be exploited in wound healing. The complex interplay of all these factors must be fundamentally understood in order to establish relationships through experiments at the buffer–nano–bio interface. Therefore, as a first step, it is necessary to systematically prepare and investigate biocompatible AuNPs with tuneable properties with respect to influencing parameters such as pH and *I_c_* and to establish meaningful simulation models to study the influence of these conditions at the NP surface.

For this purpose, a robust manufacturing strategy is required, yielding AuNPs with defined initial properties. In general, AuNPs are produced in a batch process using different synthesis methods, such as chemical reduction, plant-assisted synthesis (green synthesis) or with the continuous synthesis in flow reactors [25,26,27]. Chemical synthesis methods include the Turkevich and Brust method, the seed-mediated growth and digestive ripening method [28,29]. Thereby, the Turkevich method is the most commonly used one-step preparation method, which allows the production of a broad range of spherical AuNP by simply adjusting the reducing agent ratio. The most studied reducing agents for this purpose are ascorbic acid, citrate, UV light and amino acids [30,31,32,33]. The Brust method, on the other hand, is also a simple strategy but limits the biological application of NPs produced, and the seed-mediated growth method allows the manufacturing of rod-shaped structures. The digestion ripening method is also a simple fabrication method of monodisperse NPs; however, it is difficult to control particle shape because very high temperatures are required [34]. To overcome environmental limitations, green synthesis has gained more and more attention in recent years. This synthesis strategy is also reported to be a simple, straightforward and cost-effective process in which shape and size can be regulated [35]. Moreover, enhanced bioactivity can be achieved, leading to an apoptotic effect in cancer cells [36]. The difficulty here, however, is that the number of organic components is very high, making it difficult to accurately identify the relevant reactive components.

Based on the advantages and disadvantages of the different production methods, the Turkevich method was chosen in this study to produce spherical AuNPs of different sizes.

To ensure reproducible particle production, the design-of-experiments (DoE) approach was applied. After a risk assessment, the most influential process and formulation parameters were identified, methodically reviewed and correlated. The AuNPs used in this study were considered “bare” since they were coated only with the reductant in order to understand their behaviour without introducing another coating agent that might alter the fundamental surface properties. In a further step, the prepared purified AuNPs were carefully characterized under defined conditions, i.e., dispersed in an aqueous solution of hydrochloric acid/sodium chloride (HCl/NaCl) at an initial pH ≈ 3 and different *I_c_*. In the next step, particle behaviour studies were performed with buffer systems adapted for wound treatment, mimicking both the pH and relevant *I_c_* (i.e., therapeutically relevant or high). For this purpose, lactate (LA/NaL) and citrate (H_3_Cit/Na_3_Cit) buffers, as well as Sørensen’s phosphate buffers (PBS), were used as potential dispersants. Characterization methods included size, zeta potential, shape, and (single NP) SPR using dynamic light scattering (DLS), transmission electron microscopy (TEM), atomic force microscopy (AFM), UV-VIS spectrophotometry and extended spectroscopy. The data obtained were then used for simulation studies to predict the behaviour of the AuNP surface in the studied buffers as a function of pH (and *I_c_*).

## 2. Materials and Methods

### 2.1. Materials

Tetrachloroauric acid (HAuCl_4_·3H_2_O), trisodium citrate dihydrate (Na_3_Cit·2H_2_O), sodium chloride (NaCl), sodium hydrogen carbonate (NaHCO_3_) and hydrochloric acid (HCl, w = 36%) were purchased from Carl Roth Gmbh & Co (Karlsruhe, Germany). Ascorbic (AA) and citric acid (H_3_Cit) were obtained from Herba Chemosan Apotheker-AG (Graz, Austria). Disodium hydrogen phosphate (Na_2_HPO_4_), sodium dihydrogen phosphate (NaH_2_PO_4_) and lactic acid (LA, w = 90%) were purchased from VWR Chemicals (Leuven, Belgium). Sodium lactate (NaL, w = 50%) was purchased from Caesar & Loretz GmbH (Hilden, Germany). Sodium hydroxide (NaOH) and nitric acid (HNO_3_, w = 68%) were obtained from Merck KgaA (Darmstadt, Germany). All the chemicals were used as received without additional purification. Ultrapure water (MQ water, resistivity of 18 MΩ·cm, Millipore S.A.S., Molsheim, France) was used for the solution preparation.

### 2.2. Methods

#### 2.2.1. Synthesis of AuNPs Using Na_3_Cit and AA: Design-of-Experiments (DoE) Studies

AuNPs were prepared using two common reductants, Na_3_Cit and AA, in various molar ratios. Citrate-capped AuNPs were fabricated with the chemical reduction synthesis of HAuCl_4_ with Na_3_Cit according to the modified protocol of Dong et al. [37]. Alternatively, AA was used as the reducing agent, and particles were prepared following the protocol by Malassis et al. [38]. Briefly, for both procedures, the glassware was cleaned with aqua regia (HCl:HNO_3_ = 4:1). For the preparation of the citrate-capped AuNPs, 5 mL of 0.25 mM HAuCl_4_ was heated to 100 °C under constant stirring on a magnetic stirrer, and different amounts of 34 mM Na_3_Cit were added. The synthesis was considered complete when the colour of the suspension stopped changing; the suspension was cooled to room temperature (RT). For the synthesis with AA, 5 mL of 0.5 mM HAuCl_4_ were mixed with different amounts of 0.1 M AA, and agitated on a magnetic stirrer at RT.

To optimize the syntheses and develop a predictive model, a DoE study was performed. MODDE^®^ software (Version 13.0, Sartorius AG, Göttingen, Germany) was used, and the central composite face-centred (CCF) quadratic experimental design was selected. For the screening of the reduction synthesis with Na_3_Cit, the molar ratio of Na_3_Cit to HAuCl_4_ (1.5:1 to 3.7:1), the process temperature (70–100 °C), the pH of the reaction media (3–6), the stirring speed (150–300 rpm) and the reaction time (5–90 min) were selected as input parameters. The resulting particle sizes, polydispersity indices (PdIs) and zeta potential values were used as responses. With the experimental design chosen, MODDE^®^ proposed a total of 29 experiments, 3 of which were used to investigate reproducibility. For the synthesis process involving AA, input parameters such as the molar ratio of AA to HAuCl_4_ (0.8:1 to 2.5:1), the pH of the reaction media (3–8), the stirring speed (150–300 rpm) and the reaction time (2–60 min) were tested with regard to their influence on the resulting particle size, yield and zeta potential. For this, 27 experiments in total (with 3 centre point runs for reproducibility studies) were required, and the mean values of responses were used for statistical analysis using multiple linear regression (MLR) (MODDE^®^). Coefficients plots, the summary of fit including R^2^ (i.e., percent of the variation of the response explained by the model) and Q^2^ (i.e., percent of the variation of the response predicted by the model according to cross-validation) values, the residuals’ normal probability and plots of observed vs. predicted values were used to investigate and evaluate the obtained model.

Subsequently, optimized suspensions were prepared according to the results obtained via DoE and were dialyzed for 24 h in MQ water using a dialysis membrane (Carl Roth GmbH & Co, Karlsruhe, Germany) with a molecular weight cutoff from 12 to 14 kDa, and the media was changed twice.

#### 2.2.2. Physicochemical and Optical Characterization of AuNPs

The particles were characterized regarding hydrodynamic particle size (*d*_h_) and zeta potential (ζ) via DLS and electrophoretic light scattering (ELS) after dialysis. For DLS and ELS measurements, the Zetasizer Nano ZS (Malvern, UK) was used with a He–Ne red-light-emitting laser (wavelength, λ = 633 nm). The AuNP suspensions were diluted 1:10 with prefiltered MQ water (Whatman filter, pore size 0.02 μm) to avoid multiple scattering. All measurements were performed in triplicate at RT with the samples remaining in equilibrium for 2 min, considering the refractive indices of AuNPs (0.2) and the dispersant (1.33). During the DLS studies, scattered light was detected at an angle of 173° (i.e., backscatter mode), and data processing was performed with the Zetasizer software 6.32 (Malvern Instruments). The obtained data are presented as volume-based particle-size distribution to avoid overestimation of larger particles due to higher scattered light intensities. The zeta potential of the AuNPs was calculated from the measured electrophoretic mobility by applying the Henry equation using the Smoluchowski approximation (*f*(κa) = 1.5).

The morphology of AuNPs was determined with TEM measurements. AuNP suspensions, diluted in a 1:1 ratio, were placed on carbon-coated TEM grids and blot dried. Grids were visualized with a Thermo Fisher Tecnai 20 transmission electron microscope operating at 120 kV acceleration voltage. Microscopic images were taken with a Gatan US 1000 CCD camera.

To verify the measured sizes and investigate the shape of the synthesized particles, visualization of AuNPs was performed with atomic force microscopy (AFM; FlexAFM atomic force microscope equipped with an Easyscan 2 controller, Nanosurf, Liestal, Switzerland). For the AFM studies, 10 μL of each AuNP suspension was placed on a silicon wafer and dried overnight at RT. Prior to use, the wafer was rinsed with deionized water and ethanol and flushed with nitrogen. Noncontact (tapping) mode with a setpoint of 60% was used for the acquisition using a silicon nitride tip (Tap300Al-G; Budgetsensors, Sofia, Bulgaria) with a radius of 10 nm and a length of 125 µm. Samples were screened with a nominal spring constant of 40 N/m and a nominal resonance frequency of 300 kHz at ambient temperature. Images were processed with Gwydion Data Processing Software (Version 2.62) [39]. Sizes of AuNPs were obtained by using ImageJ software (Version 1.53p) and analysis of 15 single particles from TEM images and 30 single particles from AFM images.

UV-Vis spectrophotometry measurements (Eppendorf BioSpectrometer^®^ kinetic, Darmstadt, Germany) were conducted to study the SPR of AuNPs. Samples were diluted in a 1:1 ratio and the measurements were performed over a λ range from 400 to 800 nm.

#### 2.2.3. Effect of pH and Ion Concentration on the Physicochemical and Optical Properties of AuNPs

To investigate the effect of pH and ion concentration on changes in AuNPs in terms of zeta potential, agglomeration tendency and SPR, particles were dispersed in HCl/NaCl aqueous solutions and buffer systems for wound treatment. For the HCl/NaCl aqueous solutions, pH titrations were conducted with the titrant NaOH (0.1 M) at 2 *I_c_* and 10 and 50 mM (adjusted with HCl and NaCl). pH was recorded with a pH meter (Lab 860 pH meter; Fisher Scientific GmbH, Vienna, Austria) equipped with a combined pH electrode (SI Analytics GmbH, Mainz, Germany). pH titrations were performed in the direction from acid to base. For the adjusted buffers, LA/NaL (pH 2.8–4.8), H_3_Cit/Na_3_Cit buffer (pH 2.8–5.8) and PBS (pH 6.8–8.0) at two *I_c_*, the same as for pH titrations, were prepared and the pH was recorded. 100 µL of each AuNP dispersion was incubated with 900 μL of each buffer system for 5 min, and the pH was monitored throughout the experiments. The size and zeta potential measurements were performed with the same setups as described in Section 2.2.2.

For SPR measurements, the particles were incubated with HCl/NaCl aqueous solutions and the respective buffers. To gain better insight into the optical properties of AuNPs and SPR behaviour in media with different compositions, an extended spectroscopic method was applied, which makes it possible to examine individual particles with regard to the SPR. A confocally aligned optical setup [40,41] was utilized, which featured a high numerical aperture (NA = 0.9) objective to focus a linearly polarized Gaussian beam onto the sample. The sample of interest was mounted to a 3D piezo-driven positioning stage, which provided a precision of roughly 2 nm. A 1.3 NA oil immersion objective collected the transmitted light from the sample, which was then propagated onto a photodiode. The extreme focusing, in conjunction with the precise positioning stage, enabled one to optically probe an individual NP of interest while avoiding unwanted near-field excitations. A λ sweep was performed, and the transmitted power was recorded in order to measure the resonant properties of the individual AuNPs.

#### 2.2.4. Simulation of the Local pH-Value Distribution near the Particle Surface

The pH-value distribution near the NPs was derived from the set of spherical diffusion equations for each mobile species in the solution (Equation (1)). Due to the fast kinetics of ionic reactions in solution, all species can be assumed to be in local thermodynamic equilibrium [42]. This allows one to link the equations and express the concentration of the involved species with a pH gradient. Details of the mathematical derivation are reported elsewhere [23]. For simplicity, it is assumed that the particles are spherical.

(1) $$\frac{\partial c_{i}}{\partial t}=\frac{1}{r^{2}}\mathrm{div}\left(r^{2}D_{i}\nabla c_{i}+r^{2}\frac{D_{i}z_{i}e_{0}}{k_{B}T}c_{i}\nabla \phi \right),$$

where the index *i* denotes the chemical species (H^+^, OH^−^ and the buffer ions), *c* the concentration, *D* the diffusion coefficient, *r* the radial distance from the centre, *z* the electric charge, *e*_0_ Coulomb’s constant, *k_B_* Boltzmann’s constant and *ϕ* the electrical potential. Due to the short diffusion pathways of only a few µm, the set of partial differential equations can be solved for steady-state conditions, as further described in [23] for the case of flat surfaces and NP geometries [24]. Given the high *I_c_* of the solution, the gradient of the electric potential is small, and the migration term in Equation (1) can be simplified to the case of diffusion only. The effect of an electric field will lead to a transition of the pH gradient towards/away from the NPs without changing the main shape of the pH distribution.

Results are displayed as a 2-dimensional contour plot of the local pH value as a function of distance from the electrode surface (*x*-axis) and buffer concentration (*y*-axis) for a given nanoparticle size.

#### 2.2.5. Statistics

All experiments were performed in triplicate if not stated otherwise, and mean values, including standard deviations, are presented. To evaluate the statistical significance of the size of the particles, *I_c_* and pH values of buffers, a three-way analysis of variance (ANOVA) using the Tukey test (GraphPad Prism 8, La Jolla, CA, USA) was applied. The significance of the results is indicated according to *p*-values: * *p* ≤ 0.05, ** *p* ≤ 0.01, *** *p* ≤ 0.001 and **** *p* ≤ 0.0001. The *p*-value below 0.05, i.e., 95% of the confidence interval, was considered statistically significant.

## 3. Results

### 3.1. Design-of-Experiments (DoE) Studies

**Figure 1:**
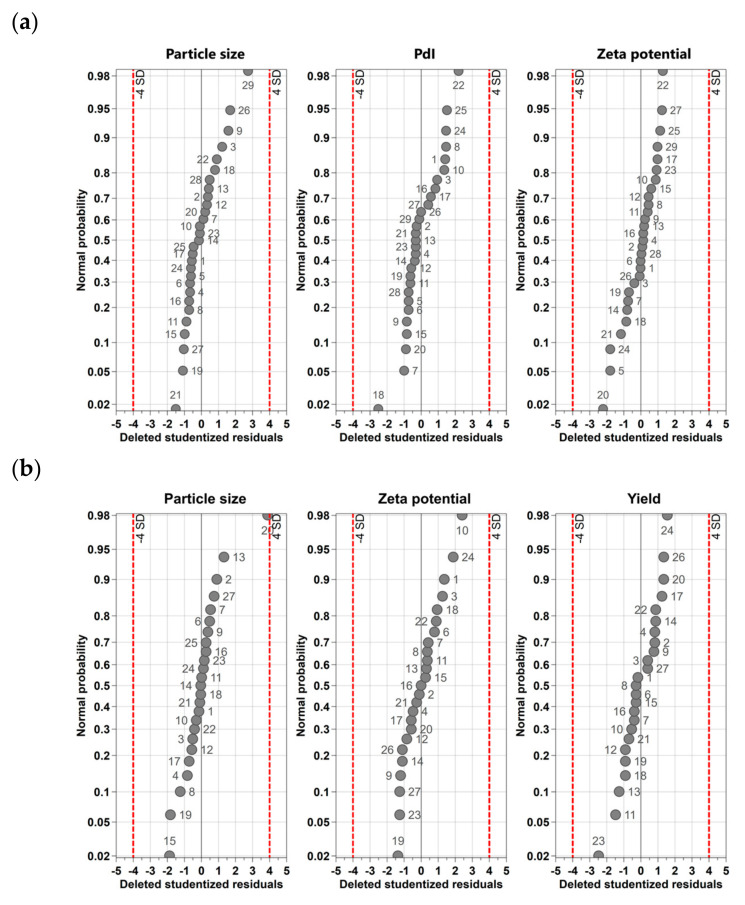
Normal probability plots of the standardized effects shown for (**a**) citrate-capped AuNPs and (**b**) AA-capped AuNPs.

One of the most common batch synthesis methods for the preparation of AuNPs is the Turkevich method, which chemically reduces metal salts in aqueous media using suitable, mild reducing agents such as Na_3_Cit [43,44] or AA [38,45]. In this process, the molar ratio of the reducing agent and metal salt is a crucial parameter that determines the particle size and PdI. Other parameters that need to be considered are pH, temperature, reaction time and the presence of other ions and molecules [46,47,48,49]. To optimize the procedure, a DoE study was performed to adjust the aforementioned parameters for both reduction agents. The obtained data, using Na_3_Cit as a reduction agent for particle size, were in the range of 5.95 ± 2.26 nm and 45.91 ± 3.14 and showed PdI values between 0.07 ± 0.01 and 0.75 ± 0.29. The zeta potential values ranged from –35.53 ± 0.58 mV to 0.90 ± 0.16 mV for the prepared batches. The use of AA as a reduction agent resulted in a larger *d*_h_ with mean sizes ranging from 44.71 ± 2.99 nm to 212.33 ± 2.45 nm. DLS measurements proposed rather polydisperse samples with at least two fractions, i.e., one from 40 to 70 nm and the second between 150 and 180 nm. This may indicate agglomeration rather than the formation of larger particles. To carefully identify the most appropriate synthesis conditions yielding de-agglomerated and thus more stable batches, the focus was laid on the fraction of the smallest sizes around 40 and 70 nm by including the respective yield. The yield for the small fraction ranged from 4.5 ± 0.0% to 77.63 ± 19.43%. At specific process conditions, the small fraction disappeared and was replaced by larger agglomerate assemblies with 100% yield. The experimental data were further used for statistical data analysis. The residuals’ normal probability plots were created (Figure 1a (Na_3_Cit) and Figure 1b (AA)) to evaluate whether the process setup and measurement strategy were reliable and to identify potential outliners (i.e., experiments outside or close to the boundaries (dashed lines)).

**Figure 2:**
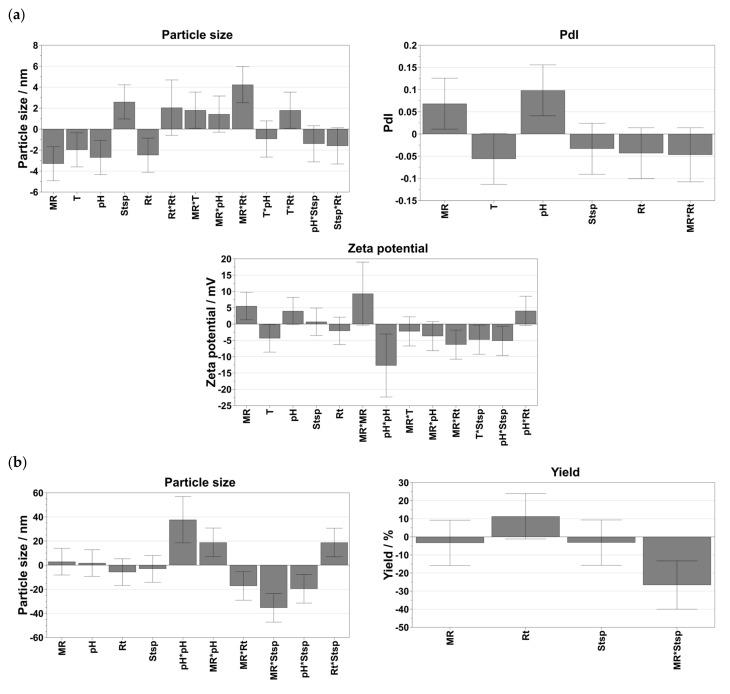

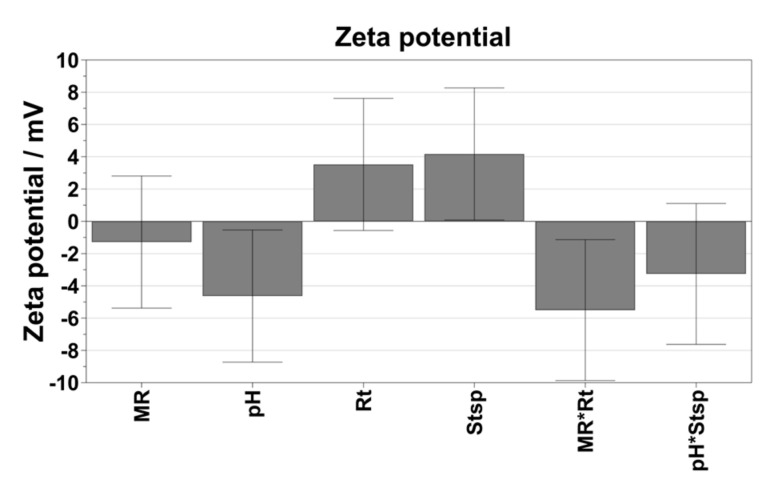
Coefficients plots shown for (**a**) citrate-capped AuNPs and (**b**) AA-capped AuNPs. MR = molar ratio, temperature = T, stirring speed = Stsp, reaction time = Rt. The asterisk in each case indicates the interaction between two tested parameters.

For Na_3_Cit, the results of all performed experiments were found to be normally distributed regardless of the considered response (i.e., size, PdI, zeta potential), as all data are on a straight line. By contrast, the use of AA as a reduction agent revealed some potential outliners. Considering the size of the response, experiments N5 and N26 were excluded as they were outside the boundaries. For the zeta potential and the PdI, the results of run N5 and N25 were excluded from further statistical data evaluation, as they were also outside the boundaries. Coefficients plots were created to assess the significance of the coefficients and are shown in Figure 2a (i.e., citrate-capped AuNPs) and Figure 2b (i.e., AA-capped). After excluding insignificant parameters (i.e., a small distance from y = 0 and error bars crossing y = 0) to simplify the model and to maximize the performance of prediction, all individually tested input parameters (i.e., molar ratio, pH, reaction time, stirring speed and temperature) showed a significant influence on the resulting particle sizes when using Na_3_Cit (Figure 2a). In addition, the combinations of molar ratio and reaction time and reaction time and temperature also affected the sizes significantly. The PdI was mainly influenced by the molar ratio, temperature and pH. The zeta potential was affected by the molar ratio, the pH, the reaction temperature and the quadratic effects of the pH and the molar ratio. In addition, the interaction in terms of the molar ratio and reaction time, and to a lower extent, the temperature and stirring speed, as well as the pH and stirring speed, strongly influenced the resulting zeta potential. During the synthesis of AuNPs via AA, size was controlled by the quadratic effects of pH and with the combination of molar ratio and pH, molar ratio and reaction time, molar ratio and stirring speed and reaction time and stirring speed. The zeta potential was significantly influenced by the pH of the reaction media, the stirring speed and the combination of molar ratio and reaction time. Considering the yield of the smallest particle fraction in the batches, it was observed that the combinatory effect of molar ratio and stirring speed had the highest impact.

For quality assessment of the model, the obtained data were fitted using MLR, and the summary of fit was prepared considering R^2^ and Q^2^ values as well as the model validity and reproducibility. For both synthesis strategies, values for R^2^ and Q^2^ were highest for the particle sizes. With comparable high R^2^ values (i.e., 0.88 for Na_3_Cit and 0.89 for AA), the variability in the data can be explained by the model. As for both strategies, Q^2^ values are above 0.5 (i.e., 0.57 for Na_3_Cit and 0.72 for AA), and the difference between R^2^ and Q^2^ is less than 0.3; the precision and reliability of future prediction can be considered high. However, it must be emphasized that the reproducibility of both batch-based synthesis routes must be classified as rather low. In the synthesis with AA, obvious outliners were already identified and excluded during the statistical data analysis step. When using Na_3_Cit in the synthesis, the results of the reproducibility tests already show a large variability in the results. Moreover, reliable predictions for zeta potential and PdI or yield can only be expected to a moderate extent for both synthesis strategies. Although all of the obtained R^2^ values were above 0.5 (i.e., 0.57 for PdI and 0.78 for the zeta potential with Na_3_Cit and 0.59 for zeta potential and 0.52 for the yield AA), which indicates an acc fit between the variability observed and the regression model; all Q^2^ values were found to be below 0.5. The experimental data show a correspondingly inhomogeneous character of some batches with high PdI values (Na_3_Cit) and a pronounced polydisperse particle size distribution (AA). Since the zeta potential is a size-dependent property and is therefore difficult to capture in polydisperse systems, the established models will primarily be used to produce AuNPs with the desired sizes as homogeneously as possible. With the optimized synthesis conditions, two different size ranges of AuNPs were prepared. Briefly, a molar ratio of Na_3_Cit:HAuCl_4_ of 3.7:1 at pH 3.3, a synthesis temperature of 100 °C at 250 rpm and reaction duration of 5 min, resulted in approximately 18 nm sized particles, referred here as “small particle size”. The use of a 1.2:1 AA:HAuCl_4_ molar ratio at pH 3.5 and 250 rpm and a reaction duration of 2 min resulted in about 56 nm, referred to here as “medium particle size”. In particular, the results after dialysis of the nanosuspensions showed that a monomodal size distribution with a d_h_ of 17.65 ± 0.38 nm was achieved with the reducing agent Na_3_Cit. When AA was used as the reductant, 56.02 ± 0.58 nm sized particles were obtained. As expected, the zeta potential values of produced AuNPs were −22.80 ± 2.50 mV and −37.60 ± 1.30 mV, respectively.

### 3.2. Physicochemical and Optical Characterization of AuNPs

**Figure 3:**
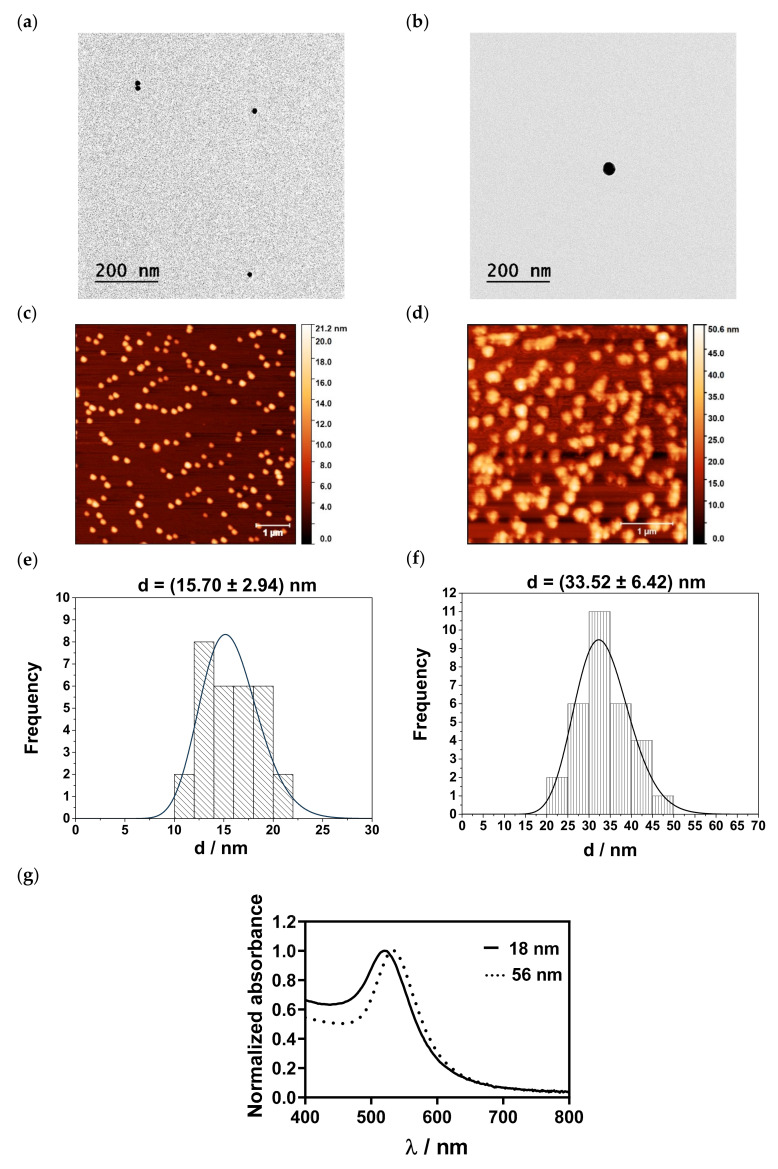
AuNPs characterization: (**a**,**b**) TEM micrographs, (**c**,**d**) AFM micrographs, (**e**,**f**) histograms of the AuNP size distributions obtained from AFM images and (**g**) optical properties of AuNPs of different sizes conducted with UV-Vis spectrophotometry. (**a**,**c**,**d**) 18 nm AuNPs; (**b**,**d**,**f**) 56 nm AuNPs.

The shape and size of the AuNPs were determined with TEM and AFM. Representative TEM images are shown in Figure 3a,b. Citrate-capped AuNPs were spherical and had a size of 14.57 ± 2.00 nm, while AA-capped AuNPs exhibited a size of 39.02 ± 5.36 nm. AFM images and histograms of the size distribution of the AuNPs are shown in Figure 3c–f. The fabricated small AuNPs were spherical in shape with a mean size of 15.70 ± 2.94 nm. AuNPs synthesized with AA exhibited sizes of 33.52 ± 6.42 nm. However, with increasing AuNP sizes, spherical clusters of AuNPs were noticed.

The optical properties, i.e., the changes of the SPR of the differently sized AuNPs, were studied with UV-Vis spectrophotometry (Figure 3g). The results showed that the changes in sizes correlated with a red shift of the SPR peak, i.e., from 520 nm for the small AuNPs to 535 nm for the medium-sized AuNPs’ fractions.

### 3.3. Effect of the pH on the Physicochemical and Optical Properties of AuNPs

**Figure 4:**
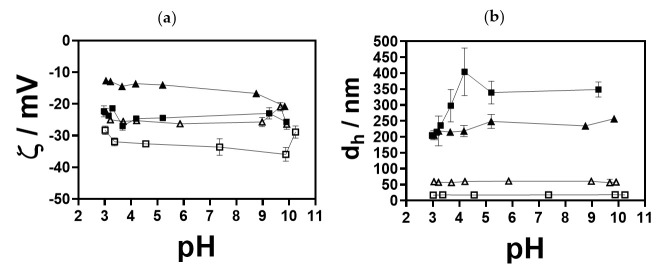
pH-dependent (**a**) zeta potential and (**b**) size profiles of AuNPs of different sizes in HCl/NaCl aqueous solution. pH titrations were conducted with the titrant NaOH (0.1 M) at two *I_c_*, i.e., 10 and 50 mM (adjusted with HCl and NaCl); (□) 18 AuNPs at *I_c_* = 10 mM, (■) 18 nm AuNPs at *I_c_* = 50 mM, (△) 56 nm AuNPs at *I_c_* = 10 mM and (▲) 56 nm AuNPs at *I_c_* = 50 mM.

The changes of the zeta potential and size of the small- and medium-sized AuNPs as a function of the pH at two nearly constant *I_c_*, i.e., 10 and 50 mM, are presented in Figure 4a,b. The results showed that, with increasing pH, the zeta potential of the small-sized AuNPs remained constant at 10 mM *I_c_* (−28.33 ± 1.35 mV (pH = 3.0) to −28.87 ± 1.97 mV (pH = 10.2)).

**Figure 5:**
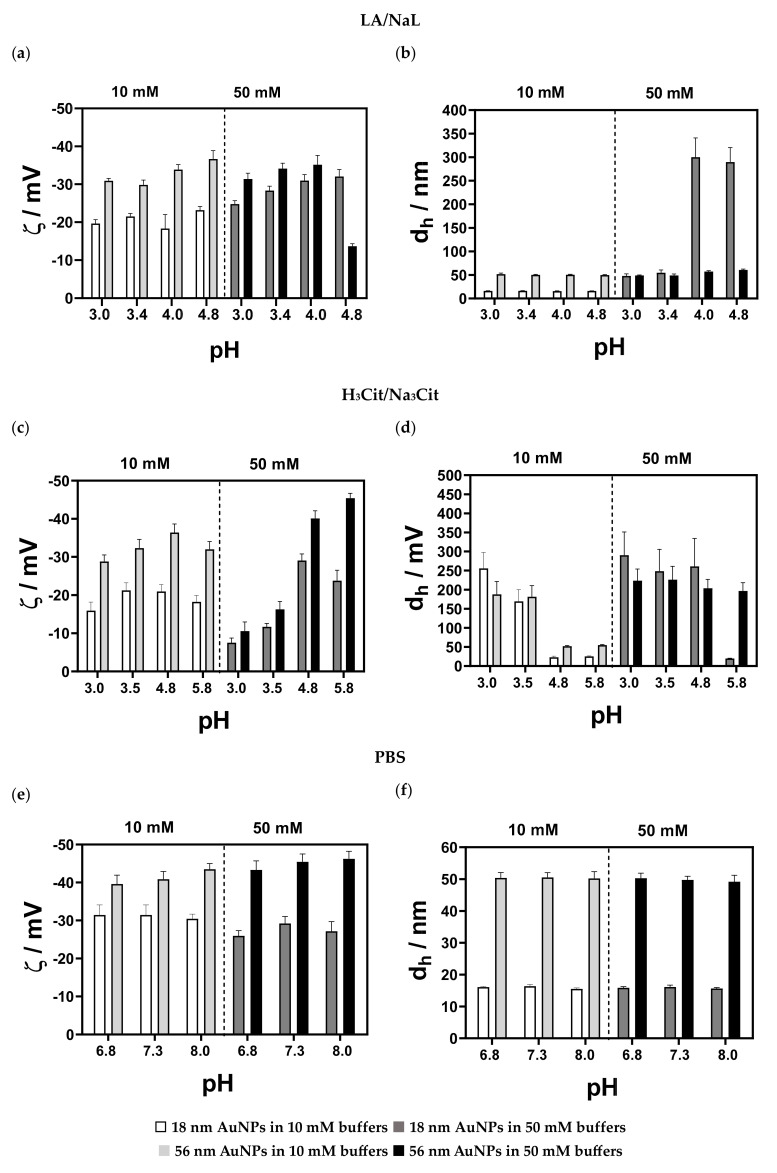
(**a**,**c**,**e**) Zeta potential and (**b**,**d**,**f**) size profiles of 18 nm and 56 nm sized AuNPs, in (**a**,**b**) LA/NaL buffer (pH = 2.9–4.8), (**c**,**d**) H_3_Cit/Na_3_Cit buffer, pH = 2.8–5.8) and (**e**,**f**) PBS (pH = 6.8–8) at two *I_c_*, 10 mM and 50 mM. The dashed line in each case separates the datasets of 18 nm and 56 nm measured NPs in 10 mM buffer (left part) and the datasets of 18 nm and 56 nm measured NPs in 50 mM buffer (right part).

As expected, at higher *I_c_*, i.e., 50 mM, the zeta potential values were lower. The isoelectric point was not noticed, and the zeta potential remained negative over the entire pH range. pH-dependent zeta potential profiles for 56 nm sized AuNPs exhibited similar behaviour, with lower zeta potential values compared to 18 nm sized AuNPs. At 10 mM, the sizes of small and medium AuNPs were similar to the AuNPs dispersed in MQ water and remained constant in the pH range tested. The increase in *I_c_* led to an irreversible agglomeration of 18 nm and 56 nm sized AuNPs, independent of the pH. The changes in the zeta potential and size of the small- and medium-sized AuNPs in the LA/NaL-, H_3_Cit/Na_3_Cit buffers and PBS at 10 and 50 mM are presented in Figure 5a–f. In 10 mM LA/NaL buffer (Figure 5a,b), small-sized AuNPs retained their initial sizes regardless of the pH; however, as the *I_c_* increased to 50 mM, the size of AuNPs increased from 48 to 290 nm with a rising pH. At 10 mM, the zeta potential was ≈−20 mV for all pH values. In 10 and 50 mM H_3_Cit/Na_3_Cit buffer (Figure 5c,d), at lower pH values, the zeta potential of the small NPs changed, indicating that they exhibited less stability. Small-sized AuNPs in PBS (Figure 5e,f) were stable in terms of zeta potential and size at both investigated *I_c_*. The medium-sized AuNPs in LA/NaL buffer (Figure 5a,b) at pH 2.8 showed a slightly lower zeta potential at both *I_c_*. Still, the size was in the range of 50–60 nm, thus confirming a high particle stability. Incubation of the particles in 10 mM H_3_Cit/Na_3_Cit buffers (Figure 5c,d) exhibited the same behaviour as in the case of the 18 nm sized AuNPs. With increasing *I_c_*, agglomeration occurred.

The particles dispersed in 10 and 50 mM PBS exhibited high zeta potential values (ζ > −40 mV) (Figure 5e,f), and the initial particle size was preserved independent of the pH. 

A three-way analysis of variance (ANOVA) was applied to test the statistical differences between size and zeta potential measurements for small- (18 nm) and medium (56 nm)-sized AuNPs at two different *I_c_* and pH values of the buffers. There were no statistically significant differences between the AuNP sizes (*p* = 0.5433). Both *I_c_* and buffer pH significantly affected the obtained sizes (*p* ≤ 0.0001). There was a significant interaction between pairwise comparisons of *I_c_* and buffers (*p* ≤ 0.0001) and *I_c_* and the size of AuNPs (*p* ≤ 0.0001). Significance was also determined for the interaction between the size of AuNPs and *I_c_* (*p* = 0.0128). A three-way ANOVA showed that the interaction between AuNP size, *I_c_* and the pH value of buffers was statistically significant (*p* ≤ 0.0001). A similar trend was obtained for the zeta potential. It was found that the size (*p* ≤ 0.0001), *I_c_* (*p* = 0.0060) and buffer pH (*p* ≤ 0.0001) had a significant effect. For the pairwise comparison of the *I_c_* and buffer pH, there was *p* ≤ 0.001, as well as for the comparison of the AuNP size and buffer pH. The interaction among the size of AuNPs and *I_c_* was also significant (*p* = 0.0011), as was the interaction among the size of AuNPs, *I_c_*, and pH of buffers (*p* ≤ 0.0001).

**Figure 6:**
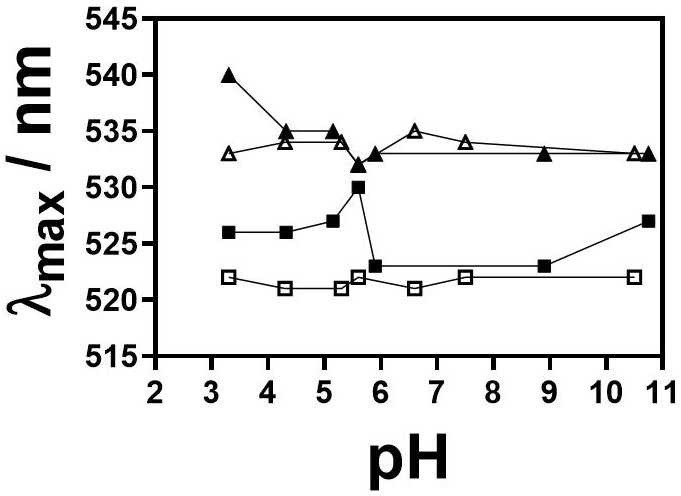
Maximum wavelength (λ_max_) of the SPR peak of 18 nm and 56 nm sized AuNPs as a function of pH obtained from UV-Vis spectra at two *I_c_*, 10 mM and 50 mM; (□) 18 AuNPs at *I_c_* = 10 mM, (■) 18 nm AuNPs at *I_c_* = 50 mM, (△) 56 nm AuNPs at *I_c_* = 10 mM and (▲) 56 nm AuNPs at *I_c_* = 50 mM.

The effects of pH and *I_c_* on the SPR peak of AuNPs were determined using UV-Vis spectrophotometry in all buffers, as shown in Figure 6. From pH 3.3 to 10.5, the small-sized AuNPs showed a red shift (Δ_max_ = 2 nm). However, the colour of the suspension remained the same. No red-wine-to-blue colour transition was noticed, suggesting that particles were stable. At 50 mM, alternations between the red and blue shift were detected (Δ_max_ = ±2 nm), while at pH = 5.6 a red shift of 3 nm was obtained. The medium-sized AuNPs exhibited the blue shift at 10 mM in the tested pH range. At 50 mM, only at pH 3.3, a red shift of 5 nm was noticed, and the colour of the suspension changed to blue. In the pH range of 4.3–10.8, the SPR peak had the same maximum as in the MQ water (535 nm) or was blue-shifted by 2 nm.

**Figure 7:**
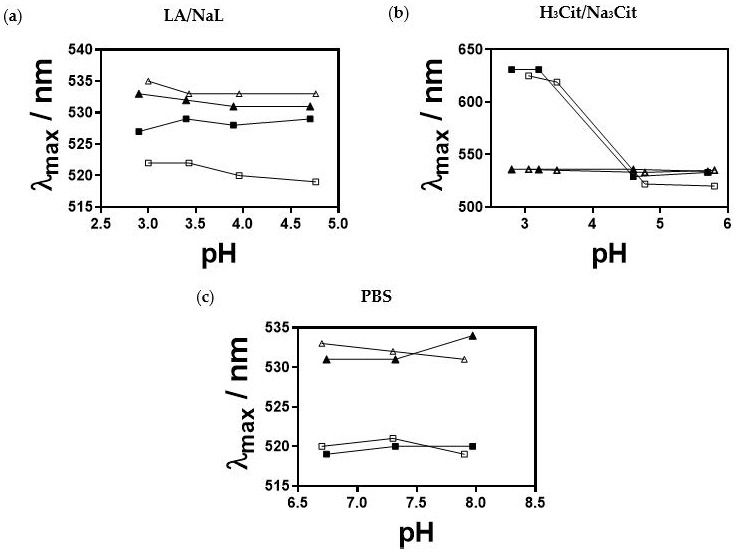
Maximum wavelength (λmax) of the SPR peak of 18 nm and 56 nm AuNPs as a function of pH obtained from UV-Vis spectra in (**a**) LA/NaL buffer (pH = 2.9–4.8), (**b**) H_3_Cit/Na_3_Cit buffer (pH = 2.8–5.8) and (**c**) PBS (pH = 6.8–8) at two *I_c_*, 10 mM and 50 mM. (□) 18 AuNPs at *I_c_* = 10 mM, (■) 18 nm AuNPs at *I_c_* = 50 mM, (△) 56 nm AuNPs at *I_c_* = 10 mM and (▲) 56 nm AuNPs at *I_c_* = 50 mM.

The SPR peaks of 18 nm and 56 nm sized AuNPs were elucidated in all buffers (Figure 7a–c). When incubated in 10 mM LA/NaL buffer, small AuNPs exhibited a negligible shift of Δλ_max_ = ± 1 nm, whereas a red shift of 7–9 nm was observed at 50 mM. This indicates that AuNPs agglomerate at increased LA/NaL buffer *I_c_*. The small-sized AuNPs showed a pronounced red shift (Δλ_max_ ≈ 100 nm) independent of *I_c_* at the pH range of 3.0 to 4.8. The colour of the suspension changed immediately from red wine to blue, indicating particle agglomeration. This was further confirmed with two peaks found in the UV spectra (second peak at λ ≈ 630 nm). There were no changes in the SPR peaks of AuNPs in 10 and 50 mM PBS. The medium-sized AuNPs in 10 mM and 50 mM LA/NaL buffers at pH ≈ 3 displayed the SPR peak at λ = 535 nm, which is comparable to the results for AuNPs dispersed in MQ water. Furthermore, the pH increase led to the blue shift (2–4 nm). For both H_3_Cit/Na_3_Cit buffer *I_c_*, the shift was ± 1 nm. The blue shift from 2 to 4 nm was also observed for AuNPs in PBS.

The change in λ_max_ of the SPR indicates that different pH conditions affect the optical properties of the AuNPs. However, to better understand the behaviour of AuNPs in various investigated systems, more advanced experiments were conducted by individually probing single AuNPs at pH 3, 5.6 and 7.4, using buffers at high *I_c_* following the method outlined in Section 2.2.3. Dark-field microscopy measurements were first performed to locate isolated AuNPs that would be selected for the resonance measurements. With a region of interest specified, the sample was then placed in an optical setup equipped for the single particle illumination scheme. At pH 7.4, several of the isolated AuNPs measured showed in the transmission spectra significant dips in λ between 546 and 591 nm. The variance in resonance can be associated with the standard deviation in the size of the AuNPs. At pH 5.6, no individual particles but, rather, nonspherical clusters were present. The respective transmission curves were broadened with no clear resonant wavelength; therefore, a resonance shift could not be assigned. The broadening of the resonance for this pH can be attributed to particle agglomeration, lack of spherical uniformity, and near-field interactions with neighbouring nanoclusters. For AuNPs prepared in a solution with pH 3.0, a broadened, yet identifiable, resonance occurred, which spanned 573–625 nm. The pH 3.0 AuNPs were also heavily agglomerated; however, the nanoclusters were more isolated, minimizing the effect of near-field interactions and broadening their respective resonance properties. Ultimately, the single-particle excitation schemes revealed that for a lower pH, the SPR resonance is red-shifted, primarily due to agglomeration. The significant agglomeration incurred is linked to the high buffer concentration used to prepare these particular samples.

### 3.4. Simulation of the Local pH-Value Distribution near the Particle Surface

**Figure 8:**
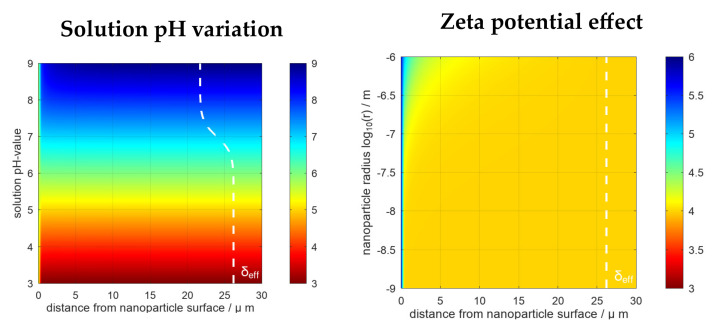
Calculated local pH-value distributions in the diffusion zone near the surface of 18 nm sized AuNPs. The default values for the simulations were 34 mM H_3_Cit/Na_3_Cit solution (solution pH = 4) and a zeta potential value of −22.8 mV (corresponding to that of the citrate capped).

The local pH values near the surface of the 18 and 56 nm sized AuNPs were determined in La/NaL, H_3_Cit/Na_3_Cit and PBS buffer at pH = 4. Figure 8 shows the effect of the solution pH variation and the influence of the zeta potential on the size-dependent local pH distribution at, for example, −22.8 mV (for other conditions, see Figures S1–S3 in Supplementary Materials). The simulation of the near surface pH-value distribution around 18 nm and 56 nm AuNPs showed very little difference. The same was noticed for different buffer systems (see Figure S4 in Supplementary Materials). It should be noticed that differences between the buffer systems become visible for more extreme pH values. These extreme cases, however, contradict the measured pH values and surface potentials in the experiments and have, therefore, not been considered. For the buffer variation and NPs with a size below 100 nm, a steep pH gradient was observed within the first 1 µm from the NP surface.

Larger NPs showed a different behaviour, comprising smaller pH gradients and hence lower current densities in cyclic voltammetry curves, which confirms that smaller particles are more reactive than larger ones. 

## 4. Discussion

One of the most widely used methods for the preparation of AuNPs is the Turkevich method, i.e., chemical reduction synthesis with the assistance of Na_3_Cit, which includes two mechanistic steps: first, electron transfer, and second, reduction with acetone [50]. Once AuNPs are formed, citrate ions physically adsorb onto their surface, stabilizing the NPs. The size of AuNPs strongly depends on the molar ratio of the reactant. More precisely, in our study, monodisperse and stable 10–20 nm AuNPs could be obtained when the molar ratio was ≥2.5. However, a further increase in the molar ratio did not affect the size, as the particle was covered with citrate ions, resulting in stabilization [51]. By contrast, the reduction of metal salts with AA includes only electron transfer; therefore, Na_3_Cit exhibits a higher redox potential than AA. Since Na_3_Cit and AA are both weak acids, their reduction potential also depends on the pH of the reaction mixture. The increase in pH leads to subsequent deprotonation and an increase in electron density, thus increasing the redox potential. By increasing the pH in the reaction mixture, the size and morphology of the final NPs can be tailored. The synthesis conditions determined in the DoE study were chosen for the fabrication of 18 and 56 nm sized AuNPs. The particle sizes determined in MQ water with DLS agreed well with the AFM and TEM measurements. AFM and TEM measurements further showed that citrate-capped AuNPs were spherically shaped. By changing the reductant, approx. 30–40 nm sized particles were measured with AFM and TEM. However, compared to the 18 nm particles, they showed a tendency to form clusters. Regarding SPR, which depends on the size, shape and surface functionalization, a shift towards a higher λ (i.e., red shift) was detected [52,53]. In addition to the size and optical properties, the zeta potential values were above −20 mV, confirming colloidal stability in MQ water independent of the pH. However, under in vivo conditions, the situation becomes more complex, which probably affects not only biological performance but also toxicity. For example, small NPs with a larger specific surface area are more reactive because more surface area is available to interact with biological molecules and cellular components [54,55,56,57,58,59]. Furthermore, the size of metal NPs can influence the release process of ions dependent on the physiological fluid and pH [60,61,62]. This suggests that pH dependence profiles of NPs are important for predicting their behaviour in more complex environments. It was found that with increasing *I_c_*, AuNPs agglomerated independently of their initial size. This is in agreement with the results of Pamies et al., who studied the stability of citrate-capped AuNPs in sodium nitrate solutions with *I_c_* ranging from 0 to 1 M [63]. They showed that with an increasing ionic concentration, agglomeration of the particles occurred, which can be attributed to the compression of the double layer that facilitates van der Waals interactions between AuNPs [64,65]. Consequently, zeta potential values are expected to decrease at higher *I_c_*, which coincides with our results. No isoelectric point was observed for both NP sizes, as the isoelectric point is reported to be between pH 2 to 4 [66]. In treatment-relevant buffers, AuNPs were susceptible to the buffer type and pH environment. The lower *I_c_* did not induce agglomeration of AuNPs in LA/NaL buffer and PBS, independent of the size. However, the H_3_Cit/Na_3_Cit buffer had a stronger effect on the zeta potential and size and altered the stability even at low *I_c_*. At 50 mM, agglomeration was noticed in both LA/NaL and H_3_Cit/Na_3_Cit buffer. Interestingly, in PBS, particles remained stable. Similar results were also found by Sangwan and Seth [67]. They monitored the stability of citrate-capped AuNPs in borate (pH = 7.5, 8.5, 9.2, 9.3), PBS (pH = 6.5, 7.5, 8.5), Tris–citrate (pH = 8.5, 9.5) and Tris–HCl (pH = 4.0, 10.5) buffer. The results confirmed that the stability of AuNPs is affected by the electrolyte composition of the buffer, pH and duration and temperature of the incubation time. To understand the behaviour of AuNPs in different buffer systems, insight into the structure of citrate and AA layers on the particle’s surface is required. Both citrate and AA ligands are known to form chelate-type complexes with metallic NPs. According to Park and Shumaker-Parry, in citrate-capped AuNPs, two carboxylate groups are in direct contact with the surface, while the third terminal carboxylate group is not bound to the surface [68]. In the case of AA, the chelating is a mixed type where a monodentate and a bidentate coordination mode of AA on AuNPs are identified [69]. Since the citrate ion is more complex in terms of chelating potential compared to AA, we expected a pronounced effect in terms of the size and zeta potential in different buffers for citrate-capped AuNPs. In LA/NaL buffer, the stabilization effect at lower *I_c_* for both AuNP sizes was achieved with the steric barrier due to the hydrogen bond network between adjacent layers of citrate or AA and lactate. The latter has an additional hydroxyl group, which increases the bonding affinity and interaction with citrate and AA. As expected, the screening effect of sodium ions at higher *I_c_* is more pronounced for citrate-capped AuNPs. In addition, agglomeration is facilitated due to the disruption of the delicate hydrogen bonding network as steric repulsions are disabled. With increasing pH, the excess of citrate-lactate layers is removed due to deprotonation and the accumulation of negatively charged lactate ions in the vicinity of the AuNP surface, causing a negative zeta potential. Regarding the H_3_Cit/Na_3_Cit, the intermolecular interaction between citrate ions that coat particles and citrate ions in the bulk are more complex. Grys et al. studied the formation of AuNP aggregates in 1 M H_3_Cit/Na_3_Cit buffer solution at pH 3.5 [70]. The surface-enhanced Raman spectroscopy revealed differences in the coordination of AuNPs with citrate ions. Because of the protonation of nonsurface-bound terminal carboxylates, citrate ions in the vicinity formed carboxylic acid dimers, and AuNPs agglomerated at lower pH values. The pH increase enabled the deprotonation of carboxylate groups, and negatively charged citrate species around AuNPs were responsible for high negative zeta potential values. On the contrary, in PBS, both AuNPs exhibited stability independent of the *I_c_*. The tendency of anions to interact with the NP surface is governed by the concentration and surface affinity of anions [71]. There are few studies that report on the competitive mechanism between citrate ions and ligands such as phosphate, amino acids, organothiols, halides and adenine [72,73,74,75,76]. White and Hjortkjaer found that phosphate anions could replace citrate ligands via oxygen atoms in phosphate anions, which resulted in phosphate-coated AuNPs [77]. The exchange between citrate and phosphate ions on the particle surface is affected by the pK values of citrate and phosphate and the ligand concentration. The concentration of phosphate species in PBS was dominant in comparison to citrate species, thus favouring the exchange between two ligands, which resulted in stable AuNPs. The obtained results suggest that the classical Derjaguin–Landau–Verwey–Overbeek (DLVO) theory, which considers the electrical double-layer interaction and attractive van der Waals force, can only partly explain our findings. Other factors that should be considered include the screening effect of monovalent cations (such as Na^+^ and H^+^) and the protective role of multivalent anions (which can co-adsorb or replace ligands on the surface of AuNPs). In addition, to fully comprehend the complex dynamics at the nano–bio interface, intermolecular interactions between ligands on the surface of AuNPs, and ligands from buffers such as hydrogen bonding, electrostatic attraction, and hydrophobic effects should be examined. The changes in the stability of AuNPs of different sizes considering the treatment-relevant pH values were also detected in the optical properties. The most pronounced SPR peak shift was found for 18 nm AuNPs incubated in H_3_Cit/Na_3_Cit buffer in the pH range 3–4. The second peak at ≈630 nm can be attributed to particle agglomeration, which, in turn, coincides with the size measurements [78].

The results obtained from simulations in the tested buffer systems of both AuNP sizes showed only small changes in the pH gradient, which proves the robustness of the synthesis method. The behaviour of AuNPs of both sizes in all tested buffers exhibited no significant differences, which might be due to the higher buffer concentration (mM) compared to changes of H^+^ and OH^−^ between the surface and solution (nM or µM). The constant pH is a consequence of small changes in the protonation of the buffer species, except for in the vicinity of the surface. The steep pH gradients near the surface indicate that the interaction of (protonated) buffer species with the NPs should be considered in future studies since zeta potential measurements (see Figure 4a and Figure 5) revealed a shift of surface pH value which the reported simulations alone cannot predict. However, simulations revealed very steep gradients less than 1µm away from the surface, where one can assume an interaction with the outer Helmholtz plane and that van der Waals interactions are likely to have an effect. In addition, steep gradients mean a high ionic flow, so smaller NPs are much more reactive than larger NPs in the upper µm range.

## 5. Conclusions

The simultaneous exploitation of wound acidification and the intrinsic properties of AuNPs represent a promising approach to wound treatment. To further advance this therapeutic concept, a fundamental understanding of the interactions at the liquid–particle interface is essential. Here, we demonstrate that AuNPs dispersed in LA/NaL buffer showed the most promising results. Particles are stable at therapeutically relevant *I_c_* regardless of their size and the reducing agent used. With an increasing *I_c_*, the citrate-capped particles agglomerate due to the shielding effect of the sodium ions and the disruption of the hydrogen bonding network. In contrast, particles dispersed in H_3_Cit/Na_3_Cit buffer agglomerate at high and low *I_c_* regardless of their size and the reducing agent used. In PBS, the particles are stable independent of their size, the reducing agent used and the *I_c_*. PBS mimics physiological pH rather than supporting a wound-healing effect but still prevents progressive alkalinization and, thus, bacterial growth. Interestingly, the modelling studies show a steep pH gradient in close proximity (<1 µm) to the NP surface under all conditions, exclusively for particles smaller than 100 nm. Steep gradients also indicate a high ionic flow, so smaller NPs are much more reactive than larger NPs, which could further promote the healing potential due to a more acidic particle surface environment but could also be beneficial in drug loading. Thus, maintaining the colloidal stability of AuNPs in acidifying buffer systems is of utmost importance. It should be noted, however, that in the next step, the protonation level of chemical species such as amino acids and buffer ions must be quantified to understand how these molecules interact with the NP surface and possibly affect colloidal stability.

## Data Availability

Not applicable.

## Supplementary Materials

The following supporting information can be downloaded at: https://www.mdpi.com/article/10.3390/nano13121878/s1, Figure S1. Calculated local pH-value distributions in the diffusion zone near the surface of 18 nm (top row) and 56 nm AuNPs (bottom row). The default values for the simulations were 34 mM La/NaL buffer solution (solution pH = 4) and a zeta potential value of −22.8 mV for 18 nm and −37.6 mV for 56 nm sized AuNPs, respectively; Figure S2. Calculated local pH-value distributions in the diffusion zone near the surface of 56 nm AuNPs. The default values for the simulations were 34 mM H_3_Cit/Na_3_Cit solution (solution pH = 4) and a zeta potential value of −37.6 mV; Figure S3. Calculated local pH-value distributions in the diffusion zone near the surface of 18 nm (top row) and 56 nm AuNPs (bottom row). The default values for the simulations were 34 mM PBS buffer solution (solution pH = 4) and a zeta potential value of −22.8 mV for 18 nm and −37.6 mV for 56 nm sized AuNPs, respectively; Figure S4. Calculated local pH-value distributions in the diffusion zone near the surface of 18 nm (top row) and 56 nm AuNPs (bottom row) with buffer concentration variation. The default values for the simulations were 34 mM buffer solutions (solution pH = 4) and a zeta potential value of −22.8 mV for 18 nm and −37.6 mV for 56 nm sized AuNPs, respectively.

## References

[1] Velnar T., Bailey T., Smrkolj V. (2009). The wound healing process: An overview of the cellular and molecular mechanisms. J. Int. Med. Res..

[2] Lodhi S., Singhai A.K. (2013). Wound healing effect of flavonoid rich fraction and luteolin isolated from Martynia annua Linn. On streptozotocin induced diabetic rats. Asian Pac. J. Trop. Med..

[3] Guo S., DiPietro L.A. (2010). Factors Affecting Wound Healing. J. Dent. Res..

[4] Proksch E., Surber C., Abels C., Maibach H. (2018). Buffering Capacity. pH of the Skin: Issues and Challenges.

[5] Siddiqui A.R., Bernstein J.M. (2010). Chronic wound infection: Facts and controversies. Clin. Dermatol..

[6] Aly R., Shirley C., Cunico B., Maibach H.I. (1978). Effect of prolonged occlusion on the microbial flora, pH, carbon dioxide and transepidermal water loss on human skin. J. Investig. Dermatol..

[7] Khalilov R. (2023). A comprehensive review of advanced nano-biomaterials in regenerative medicine and drug delivery. Adv. Biol. Earth Sci..

[8] Jain P.K., Lee K.S., El-Sayed I.H., El-Sayed M.A. (2006). Calculated absorption and scattering properties of gold nanoparticles of different size, shape, and composition: Applications in biological imaging and biomedicine. J. Phys. Chem. B.

[9] Tiwari P.M., Vig K., Dennis V.A., Singh E.R. (2011). Functionalized gold nanoparticles and their biomedical applications. Nanomaterials.

[10] Bansal S.A., Kumar V., Karimi J., Singh A.P., Kumar S. (2020). Role of gold nanoparticles in advanced biomedical applications. Nanoscale Adv..

[11] Akturk O., Kismet K., Yasti A.C., Kuru S., Duymus M.E., Kaya F., Caydere M., Hucumenoglu S., Keskin D. (2016). Collagen/gold nanoparticle nanocomposites: A potential skin wound healing biomaterial. J. Biomater. Appl..

[12] Naraginti S., Kumari P.L., das Sivakumar R.K.A., Patil S.H., Andhalkar V.V. (2016). Amelioration of excision wounds by topical application of green synthesized, formulated silver and gold nanoparticles in albino Wistar rats. Mater. Sci. Eng. C.

[13] Hung Y.L., Hsiung T.M., Chen Y.Y., Huang Y.F., Huang C.C. (2010). Colorimetric detection of heavy metal ions using label-free gold nanoparticles and alkanethiols. J. Phys. Chem. C.

[14] Gu H., Ho P.L., Tong E., Wang L., Xu B. (2003). Presenting Vancomycin on Nanoparticles to Enhance Antimicrobial Activities. Nano Lett..

[15] Norman S., Stone J.W., Gole A., Murphy C., Sabo-Attwood T.L. (2008). Targeted Photothermal Lysis of the Pathogenic Bacteria, Pseudomonas aeruginosa, with Gold Nanorods. Nano Lett..

[16] Gil-Tomás J., Tubby S., Parkin I.P., Narband N., Dekker L., Nair S.P., Wilson M., Street C. (2007). Lethal photosensitisation of Staphylococcus aureus using a toluidine blue O–tiopronin–gold nanoparticle conjugate. J. Mater. Chem..

[17] Joshi P., Chakraborti S., Ramirez-Vick J.E., Ansari Z.A., Shanker V., Chakrabarti P., Singh S.P. (2012). The anticancer activity of chloroquine-gold nanoparticles against MCF-7 breast cancer cells. Colloids Surf. B Biointerfaces.

[18] Schwert G.W., Eisenberg M.A. (1949). The kinetics of the amidase and esterase activities of trypsin. J. Biol. Chem..

[19] Han K., Zhu J.Y., Wang S.B., Li Z.H., Cheng S.X., Zhang X.Z. (2015). Tumor targeted gold nanoparticles for FRET-based tumor imaging and light responsive on-demand drug release. J. Mater Chem. B..

[20] Guo B., Zebda R., Drake S.J., Sayes C.M. (2009). Synergistic effect of co-exposure to carbon black and Fe_2_O_3_ nanoparticles on oxidative stress in cultured lung epithelial cells. Part. Fiber Toxicol..

[21] Al-Jamal W.T., Al-Jamal K.T., Bomans P.H., Frederik P.M., Kostarelos K. (2008). Functionalized-quantum-dot-liposome hybrids as multimodal nanoparticles for cancer. Small.

[22] Lundqvist M., Stigler J., Elia G., Lynch I., Cedervall T., Dawson K.A. (2008). Nanoparticle size and surface properties determine the protein corona with possible implications for biological impacts. Proc. Natl. Acad. Sci. USA.

[23] Auinger M., Katsounaros I., Meier J.C., Klemm S.O., Ulrich Biedermann P., Topalov A.A., Rohwerdera M., Mayrhofer K.J.J. (2011). Near-surface ion distribution and buffer effects during electrochemical reactions. Phys. Chem. Chem. Phys..

[24] Stepan T., Tete L., Laundry-Mottiar L., Romanovskaia E., Hedberg Y.S., Danninger H., Auinger M. (2022). Effect of nanoparticle size on the near-surface pH-distribution in aqueous and carbonate buffered solutions. Electrochim. Acta.

[25] Baber R., Mazzei L., Kim Thanh N.T., Gavriilidis A. (2017). An engineering approach to synthesis of gold and silver nanoparticles by controlling hydrodynamics and mixing based on a coaxial flow reactor. Nanoscale.

[26] Damilos S., Alissandratos I., Panariello L., Radhakrishnan A.N.P., Cao E., Wu G., Besenhard M.O., Kulkarni A.A., Makatsoris C., Gavriilidis A. (2021). Continuous citrate-capped gold nanoparticle synthesis in a two-phase flow reactors. J. Flow Chem..

[27] Zhang X., Ma S., Li A., Chen L., Lu J., Geng X., Xie M., Liang X., Wan Y., Yang P. (2020). Continuous high-flux synthesis of gold nanoparticles with controllable sizes: A simple microfluidic system. Appl. Nanosci..

[28] Turkevich J., Cooper P.H.J. (1951). A study of the nucleation and growth process in the synthesis of colloidal gold. Discuss. Faraday Soc..

[29] Brust M., Walker M., Bethell D., Schiffrin D.J., Whyman R. (1994). Synthesis of thiol-derivatised gold nanoparticles in a two-phase liquid-liquid system. J. Chem. Soc. Chem. Commun..

[30] Niidome Y., Nishioka K., Kawasaki H., Yamada S. (2003). Rapid synthesis of gold nanorods by the combination of chemical reduction and photoirradiation processes; morphological changes depending on the growing processes. Chem. Comm..

[31] Kumar S., Gandhi K.S., Kumar R. (2007). Modeling of formation of gold nanoparticles by citrate method. Ind. Eng. Chem. Res..

[32] Pal A., Esumi K., Pal T. (2005). Preparation of nanosized gold particles in a biopolymer using UV photoactivation. J. Colloid Interface Sci..

[33] Wangoo N., Bhasin K.K., Mehta S.K., Suri C.R. (2008). Synthesis and capping of water-dispersed gold nanoparticles by an amino acid: Bioconjugation and binding studies. J. Colloid Interface Sci..

[34] Sau T.K., Murphy C.J. (2004). Room temperature, high-yield synthesis of multiple shapes of gold nanoparticles in aqueous solution. J. Am. Chem. Soc..

[35] Lee K.X., Shameli K., Yew Y.P., Teow S.Y., Jahangirian H., Rafiee-Moghaddam R., Webster T.J. (2020). Recent developments in the facile bio-synthesis of gold nanoparticles (AuNPs) and their biomedical applications. Int. J. Nanomed..

[36] Wang J., Liu N., Su Q., Lv Y., Yang C., Zhan H. (2022). Green Synthesis of Gold Nanoparticles and Study of Their Inhibitory Effect on Bulk Cancer Cells and Cancer Stem Cells in Breast Carcinoma. Nanomaterials.

[37] Dong J., Carpinone P.L., Pyrgiotakis G., Demokritou P., Moudgil B.M. (2020). Synthesis of precision gold nanoparticles using Turkevich method. Kona.

[38] Malassis L., Dreyfus R., Murphy R.J., Hough L.A., Donnio B., Murray C.B. (2016). One-step green synthesis of gold and silver nanoparticles with ascorbic acid and their versatile surface post-functionalization. RSC Adv..

[39] Nečas D., Klapetek P. (2012). Gwyddion: An open-source software for SPM data analysis. Cent. Eur. J. Phys..

[40] Woźniak P., Banzer P., Leuchs G. (2015). Selective switching of individual multipole resonances in single dielectric nanoparticles. Laser Photonics Rev..

[41] Banzer P., Peschel U., Quabis S., Leuchs G. (2010). On the experimental investigation of the electric and magnetic response of a single nano-structure. Opt. Express.

[42] Eigen M. (1954). Methods for investigation of ionic reactions in aqueous solutions with half-times as short as 10^−9^ sec. Application to neutralization and hydrolysis reactions. Discuss. Faraday Soc..

[43] Ji X., Song X., Li J., Bai Y., Yang W., Peng X. (2007). Size control of gold nanocrystals in citrate reduction: The third role of citrate. J. Am. Chem. Soc..

[44] Bastús N.G., Comenge J., Puntes V. (2011). Kinetically controlled seeded growth synthesis of citrate-stabilized gold nanoparticles of up to 200 nm: Size focusing versus Ostwald ripening. Langmuir.

[45] Hussain M.H., Abu Bakar N.F., Mustapa A.N., Low K.F., Othman N.H., Adam F. (2020). Synthesis of various size gold nanoparticles by chemical reduction method with different solvent polarity. Nanoscale Res. Lett..

[46] Kimling J., Maier M., Okenve B., Kotaidis V., Ballot H., Plech A. (2006). Turkevich method for gold nanoparticle synthesis revisited. J. Phys. Chem. B.

[47] Zhao L., Jiang D., Cai Y., Ji X., Xie R., Yang W. (2012). Tuning the size of gold nanoparticles in the citrate reduction by chloride ions. Nanoscale.

[48] Suchomel P., Kvitek L., Prucek R., Panacek A., Halder A., Vajda S., Zboril R. (2018). Simple size-controlled synthesis of Au nanoparticles and their size dependent catalytic activity. Sci. Rep..

[49] Yazdani S., Daneshkhah A., Diwate A., Patel H., Smith J., Reul O., Cheng R., Izadian A., Hajrasouliha A.R. (2021). Model for gold nanoparticle synthesis: Effect of pH and reaction time. ACS Omega.

[50] Rodrigues T.S., Zhao M., Yang T.H., Gilroy K.D., da Silva A.G.M., Camargo P.H.C., Xia Y. (2018). Synthesis of colloidal metal nanocrystals: A Comprehensive review on the reductants. Chem. Eur. J..

[51] Wuithschick M., Birnbaum A., Witte S., Sztucki M., Vainio U., Pinna N., Rademann K., Emmerling F., Kraehnert R., Polte J. (2015). Turkevich in new robes: Key questions answered for the most common gold nanoparticle synthesis. ACS Nano.

[52] Ou J., Zhou Z., Chen Z., Tan H. (2019). Optical diagnostic based on functionalized gold nanoparticles. Int. J. Mol. Sci..

[53] Mie G. (1908). Contributions to the optics of turbid media, particularly of colloidal metal solutions. Ann. Phys..

[54] Sukhanova A., Bozrova S., Sokolov P., Berestovoy M., Karaulov A., Nabiev I. (2018). Dependence of nanoparticle toxicity on their physical and chemical properties. Nanoscale Res. Lett..

[55] Pan Y., Neuss S., Leifert S.A., Fischler M., Wen F., Simon U., Schmid G., Brandau W., Jahnen-Dechent W. (2007). Size-dependent cytotoxicity of gold nanoparticles. Small.

[56] Li X., Hu Z., Ma J., Wang X., Zhang Y., Wang W., Yuana Z. (2018). The systematic evaluation of size-dependent toxicity and multi-time biodistribution of gold nanoparticles. Colloids Surf. B Biointerfaces.

[57] Liu W., Wu Y., Wang C., Li H.C., Wang T., Liao C.Y., Cui L., Zhou Q.F., Yan B., Jiang G.B. (2010). Impact of silver nanoparticles on human cells: Effect of particle size. Nanotoxicology.

[58] Yu K.O., Grabinski C.M., Schrand A.M., Murdock A.M.R.C., Wang W., Gu B., Schlager J.J., Hussain S.M. (2009). Toxicity of amorphous silica nanoparticles in mouse keratinocytes. J. Nanopart. Res..

[59] Lin X., Li J., Ma S., Liu G., Yang K., Tong M., Lin D. (2014). Toxicity of TiO_2_ nanoparticles to escherichia coli: Effects of particle size, crystal phase and water chemistry. PLoS ONE.

[60] Misra S.K., Dybowska A., Berhanu D., Luoma S.N., Valsami-Jones E. (2012). The complexity of nanoparticle dissolution and its importance in nanotoxicological studies. Sci. Total Environ..

[61] Brunner T.J., Wick P., Manser P., Spohn P., Grass R.N., Limbach L.K., Bruinink A., Stark W.J. (2006). In vitro cytotoxicity of oxide nanoparticles: Comparison to asbestos, silica, and the effect of particle solubility. Environ. Sci. Technol..

[62] Bian S.W., Mudunkotuwa I.A., Rupasinghe T., Grassian V.H. (2011). Aggregation and dissolution of 4 nm ZnO nanoparticles in aqueous environments: Influence of pH, ionic strength, size, and adsorption of humic acid. Langmuir.

[63] Pamies R., Ginés Hernández Cifre J., Fernández Espín V., Collado-González M., Díaz Baños F.G., García de la Torre J. (2014). Aggregation behaviour of gold nanoparticles in saline aqueous media. J. Nanopart. Res..

[64] Edwards S.A., Williams D.R. (2004). Double layers and interparticle forces in colloid science and biology: Analytic results for the effect of ionic dispersion forces. Phys. Rev. Lett..

[65] Boström M., Williams D.R., Ninham B.W. (2001). Specific ion effects: Why DLVO theory fails for biology and colloid systems. Phys. Rev. Lett..

[66] Csapó E., Sebők D., Makrai Babić J., Šupljika F., Bohus G., Dékány I., Kallay N., Preočanin T. (2014). Surface and structural properties of gold nanoparticles and their biofunctionalized derivatives in aqueous electrolytes solution. J. Dispers. Sci. Technol..

[67] Sangwan S., Seth R. (2022). Synthesis, characterization and stability of gold nanoparticles (AuNPs) in different buffer systems. J. Clust. Sci..

[68] Park J.W., Shumaker-Parry J.S. (2014). Structural study of citrate layers on gold nanoparticles: Role of intermolecular interactions in stabilizing nanoparticles. J. Am. Chem. Soc..

[69] Zümreoglu-Karan B. (2009). A rationale on the role of intermediate Au(III)–vitamin C complexation in the production of gold nanoparticles. J. Nanopart. Res..

[70] Grys D.B., de Nijs B., Salmon A.R., Huang J., Wang W., Chen W.H., Scherman O.A., Baumberg J.J. (2020). Citrate coordination and bridging of gold nanoparticles: The role of gold adatoms in AuNP aging. ACS Nano.

[71] Aubard J., Bagnasco E., Pantigny J., Ruasse M.F., Levi G., Wentrup-Byrne E. (1995). An ion-exchange reaction as measured by surface-enhanced raman spectroscopy on silver colloids. J. Phys. Chem..

[72] Afshinnia K., Baalousha M. (2017). Effect of phosphate buffer on aggregation kinetics of citrate-coated silver nanoparticles induced by monovalent and divalent electrolytes. Sci. Total Environ..

[73] Rani M., Moudgil L., Singh B., Kaushal A., Mittal A., Saini G.S.S., Tripathi S.K., Singhe G., Kaura A. (2016). Understanding the mechanism of replacement of citrate from the surface of gold nanoparticles by amino acids: A theoretical and experimental investigation and their biological application. RSC Adv..

[74] Park J.W., Shumaker-Par J.S. (2015). Strong resistance of citrate anions on metal nanoparticles to desorption under thiol functionalization. ACS Nano.

[75] Huang P.J.J., Yang J., Chong K., Ma Q., Li M., Zhang F., Moon W.J., Zhang G., Liu J. (2020). Good’s buffers have various affinities to gold nanoparticles regulating fluorescent and colorimetric DNA sensing. Chem. Sci..

[76] Perera G.S., Athukorale S.A., Perez F., Pittman C.U., Zhang D. (2018). Facile displacement of citrate residues from gold nanoparticle surfaces. J. Colloid Interface Sci..

[77] White P., Hjortkjaer J. (2014). Preparation and characterisation of a stable silver colloid for SER(R)S spectroscopy. J. Raman Spectrosc..

[78] Li S., Lui K.H., Tsoi T.H., Lo W.S., Li X., Hu X., Tai W.C.S.C., Hung H.L., Gu Y.J., Wong W.T. (2019). pH-responsive targeted gold nanoparticles for in vivo photoacoustic imaging of tumor microenvironments. Nanoscale Adv..

