# Supplementary material for: Investigation of the Influence of Wound-Treatment-Relevant Buffer Systems on the Colloidal and Optical Properties of Gold Nanoparticles
Source: Nanomaterials (Basel). 2023 Jun 17;13(12):1878. doi: 10.3390/nano13121878 (PMC10301454; doi:10.3390/nano13121878)
Supplement: Supplementary file 1 [file nanomaterials-13-01878-s001.zip › nanomaterials-2442934-supplementary.pdf]

Supplementary Materials

Investigation of the Influence of Wound-Treatment-Relevant Buffer Systems on the Colloidal and Optical Properties of Gold Nanoparticles

Atiđa Selmani [1], Ramona Jeitler [1,2], Michael Auinger [3], Carolin Tetyczka [2], Peter Banzer [4], Brian Kantor [4], Gerd Leitinger [5] and Eva Roblegg [1,2,*]

[1] Pharmaceutical Technology & Biopharmacy, Institute of Pharmaceutical Sciences, University of Graz, Universitätsplatz 1, 8010 Graz, Austria; atida.selmani@uni-graz.at (A.S.); ramona.jeitler@uni-graz.at (R.J.)

[2] Research Center Pharmaceutical Engineering GmbH, Inffeldgasse 13, 8010 Graz, Austria; carolin.tetyczka@rcpe.at

[3] Institute of Chemical Technologies and Analytics, TU Wien, Getreidemarkt 9/164, 1060 Vienna, Austria; michael.auinger@tuwien.ac.at

[4] Institute of Physics, NAWI Graz, University of Graz, Universitätsplatz 5, 8010 Graz, Austria; peter.banzer@uni-graz.at (P.B.); brian.kantor@uni-graz.at (B.K.)

[5] Division of Cell Biology, Histology and Embryology, Gottfried Schatz Research Center for Cell Signaling, Metabolism and Aging, Medical University of Graz, 8010 Graz, Austria; gerd.leitinger@medunigraz.at

* Correspondence: eva.roblegg@uni-graz.at; Tel.: +43-316-380-8888

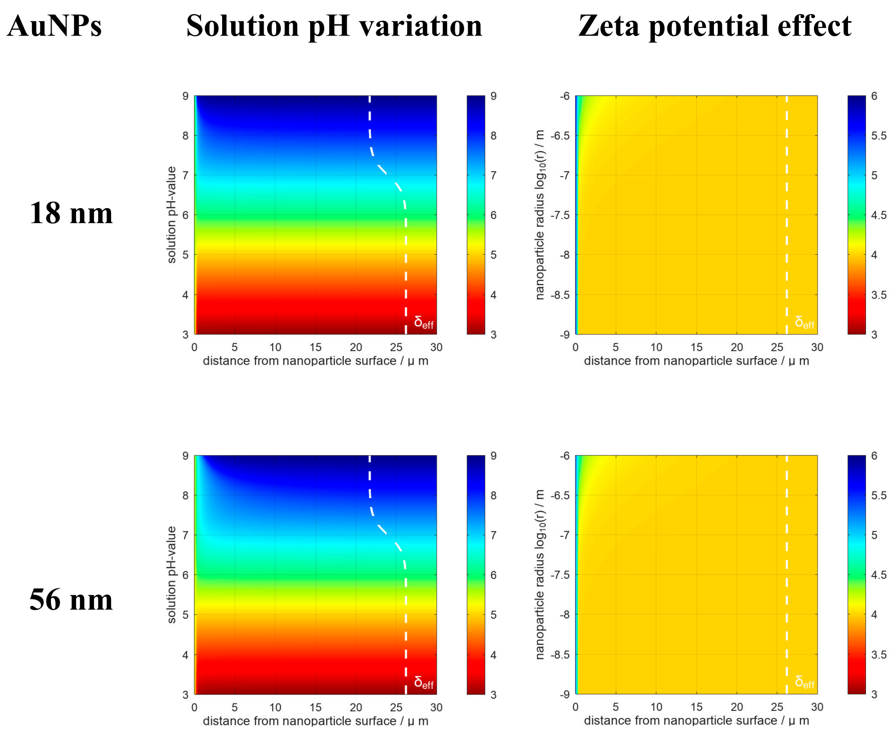

Figure S1. Calculated local pH-value distributions in the diffusion zone near the surface of 18 nm (top row) and 56 nm AuNPs (bottom row). The default values for the simulations were 34 mM La/NaL buffer solution (solution pH = 4) and a zeta potential value of −22.8 mV for 18 nm and −37.6 mV for 56 nm sized AuNPs, respectively.

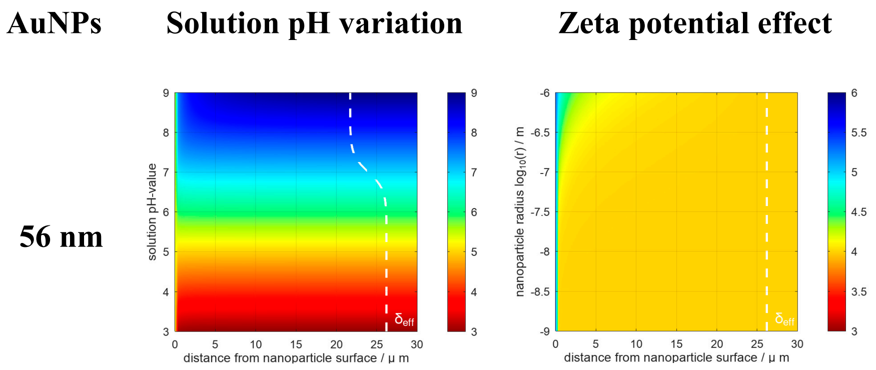

Figure S2. Calculated local pH-value distributions in the diffusion zone near the surface of 56 nm AuNPs. The default values for the simulations were 34 mM H_3Cit/Na_3Cit solution (solution pH = 4) and a zeta potential value of −37.6 mV, respectively.

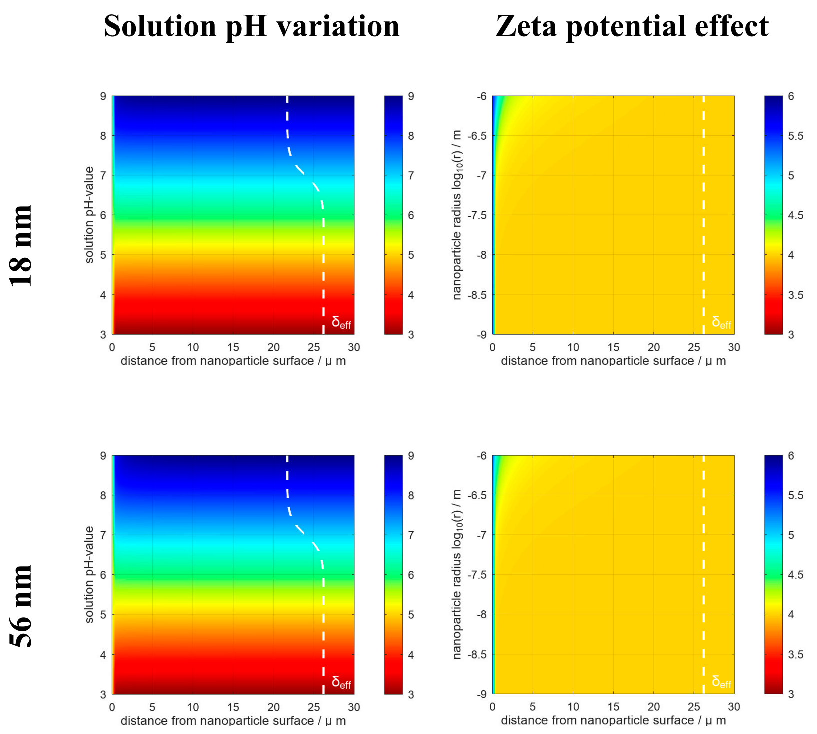

Figure S3. Calculated local pH-value distributions in the diffusion zone near the surface of 18 nm (top row) and 56 nm AuNPs (bottom row). The default values for the simulations were 34 mM PBS buffer solution (solution pH = 4) and a zeta potential value of −22.8 mV for 18 nm and −37.6 mV for 56 nm sized AuNPs, respectively.

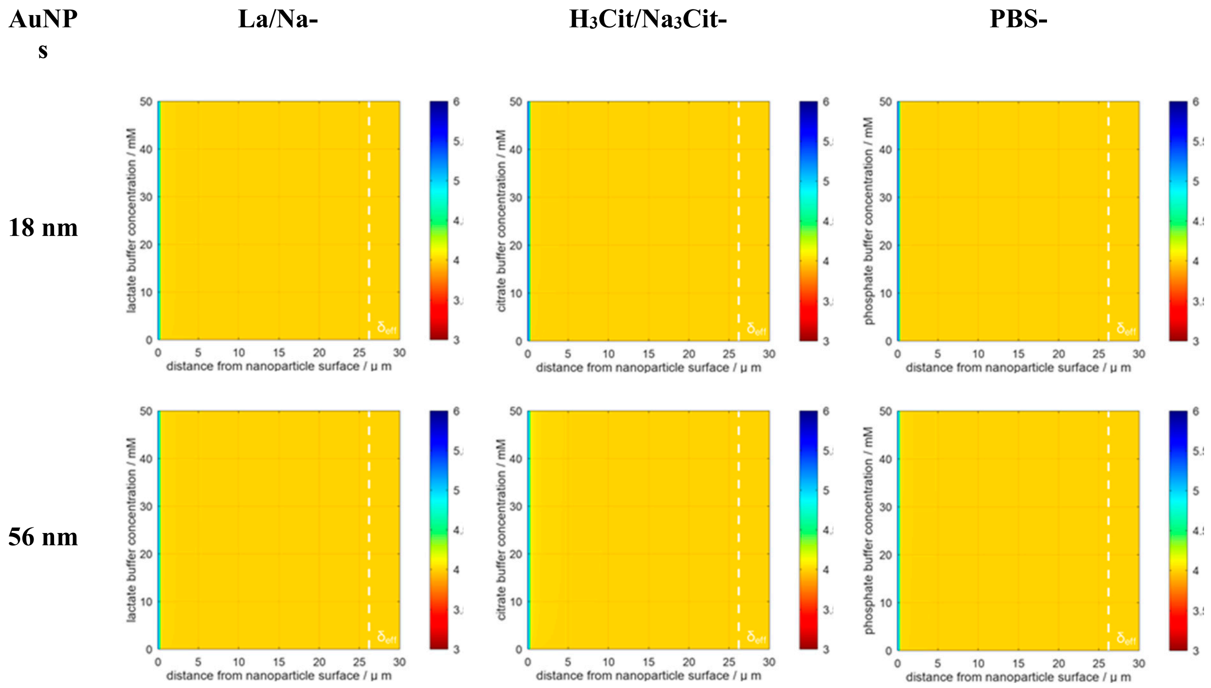

Figure S4. Calculated local pH-value distributions in the diffusion zone near the surface of 18 nm (top row) and 56 nm AuNPs (bottom row) with buffer concentration variation. The default values for the simulations were 34

mM buffer solutions (solution pH = 4) and a zeta potential value of −22.8 mV for 18 nm and −37.6 mV for 56 nm sized AuNPs, respectively.
